# The WRKY Transcription Factor GmWRKY40 Enhances Soybean Resistance to *Phytophthora sojae* via the Jasmonic Acid Pathway

**DOI:** 10.3390/biology14121769

**Published:** 2025-12-11

**Authors:** Hong Gao, Chuanzhong Zhang, Gengpu Zhang, Fengcai Guo, Yan Sun, Xin Fang, Xiaoyu Chen, Kexin Ma, Xiran Wang, Kexin Li, Jiapeng Tong, Junjiang Wu, Pengfei Xu, Shuzhen Zhang

**Affiliations:** 1 Key Laboratory of Soybean Biology of Chinese Education Ministry, Soybean Research Institute of Northeast Agricultural University, Harbin 150030, China; gaohong9@126.com (H.G.); z346797667@163.com (G.Z.); guofengcai2022@163.com (F.G.); 18846820950@163.com (Y.S.); fangxin0622@163.com (X.F.); chenx147687@163.com (X.C.); 15561851280@163.com (X.W.); likexinzhongzi@163.com (K.L.); 17832861453@163.com (J.T.); 2 College of Advanced Agricultural Sciences, Zhejiang A&F University, Hangzhou 311399, China; zhangchuanzhong92@126.com; 3 Key Laboratory of Soybean Cultivation of Ministry of Agriculture, Soybean Research Institute of Heilongjiang Academy of Agricultural Sciences, Harbin 150086, China; nkywujj@126.com

**Keywords:** soybean, *Phytophthora sojae*, WRKY transcription factor, jasmonate signaling, protein-protein interaction

## Abstract

Phytophthora root and stem rot is a destructive soybean disease worldwide, and thus improving soybean resistance to *P. sojae* is a major breeding target. However, the complex regulatory networks governing host defense remain unclear. Our previous study showed that *GmWRKY40* positively regulates resistance of soybean to *P. sojae*. Here, to explore its molecular mechanism, we found that *GmWRKY40* is induced by *P. sojae* in resistant cultivars and that the protein localizes in nucleus. RNA-seq and metabolomic analyses revealed that GmWRKY40 modulates the jasmonate (JA) signaling pathway. We then found that GmWRKY40 directly suppresses the key JA repressor *GmJAZ1* by binding to the promoter. This leads to higher endogenous JA levels, and the overall state of enhanced resistance is also characterized by elevated SOD and POD antioxidant enzyme activity. Furthermore, we demonstrated that GmWRKY40 interacts with GmWRKY36, a transcription factor identified as a negative regulator of *P. sojae* infection in this research. Taken together, our study delineates a novel regulatory module where *GmWRKY40* enhances resistance to *P. sojae* through a dual mechanism: activating the JA pathway by repressing its suppressor *GmJAZ1*, and engaging in a potentially antagonistic interaction with the negative regulator GmWRKY36, ultimately enhancing soybean resistance to *P. sojae*.

## Simple Summary

Soybean is an important global grain and oil crop, but the pathogen *Phytophthora sojae* poses a devastating threat to its yield and quality. To breed more disease-resistant soybean plants, scientists need to understand how soybeans defend against this pathogen. This study focuses on a protein named GmWRKY40 to reveal how it enhances soybean resistance to this disease. The research found that GmWRKY40 acts like a switch with a dual function. First, it activates soybean resistance to the pathogen *P. sojae* by shutting down a gene *GmJAZ1* that suppresses resistance. Second, it interacts with another protein GmWRKY36 that weakens resistance to *P. sojae*, and this interaction helps to fine-tune its defense against the pathogen. In summary, our study reveals a dual mechanism that controls soybean defense against *P. sojae*. This discovery provides breeders with precise genetic targets for developing new soybean cultivars with durable resistance to this disease, thereby helping to protect this essential food source for a growing global population.

## 1. Introduction

Plants employ a bifurcated innate immune system to counteract pathogen invasion [1]. The first tier, PAMP-triggered immunity (PTI), is initiated upon the perception of conserved pathogen-associated molecular patterns (PAMPs) by cell-surface receptors, leading to a basal defense activation [1,2]. The second, more specific tier, known as effector-triggered immunity (ETI), is mediated by intracellular R proteins that recognize pathogen effectors, often culminating in a robust hypersensitive response [1,3]. Although initiated by distinct mechanisms, both PTI and ETI converge upon a shared suite of downstream defense responses, including the production of reactive oxygen species (ROS), activation of MAPK signaling cascades and extensive transcriptional reprogramming [1,4,5]. The execution of these defenses is critically orchestrated by phytohormone signaling networks, particularly those of salicylic acid (SA) and jasmonic acid (JA) [6,7]. Typically, the SA pathway is essential for immunity against biotrophic pathogens, whereas the JA pathway is central to defending against necrotrophs, with these two hormonal branches often functioning antagonistically [8,9]. The core of the JA signaling involves the hormone JA-Ile promoting the degradation of JAZ repressor proteins via the COI1 receptor, thereby releasing transcription factors like MYC2 to activate defense gene expression [10,11,12]. These interconnected immune and hormonal signaling layers create a complex and coordinated defense system, enabling plants to resist a wide range of diseases [13].

The WRKYs are one of the largest families of plant transcription factors, characterized by a core structure comprising at least one highly conserved WRKY domain and an associated zinc finger motif [14,15]. Based on the number of WRKY domains and the type of the associated zinc finger motif, the family is classified into three main groups, and its members collectively act as central regulators in plant signaling networks [15,16,17]. First discovered in 1994 [18], their typical regulatory mechanism involves the conserved WRKY domain binding to the W-box cis-acting element in the promoters of target genes, allowing them to function as either transcriptional activators or repressors [19,20]. Within the plant immune system, WRKY factors are particularly prominent, acting as key regulatory components downstream of salicylic acid (SA) signaling elements like NPR1 and pathogenesis-related (PR) proteins to mediate resistance to pathogens [21,22]. For instance, OsWRKY13 and OsWRKY45 regulate defense against blast disease in rice [23,24]. However, their function can be dual-natured, acting as positive regulators to enhance resistance or as negative regulators (susceptibility factors) that weaken it, as seen with ScWRKY3 in sugarcane, which reduces resistance to *Ralstonia solanacearum* [25]. Furthermore, the family is also widely involved in responses to abiotic stresses such as drought and salinity [16,26,27]. The complexity of their regulatory function extends beyond direct transcriptional control, relying on protein–protein interactions (PPIs) with MAP kinases, VQ motif proteins or other transcription factors [28,29], which can finely tune the DNA-binding activity and stability of the WRKY proteins themselves [30]. Therefore, through both direct transcriptional regulation and indirect protein interaction networks, WRKY transcription factors are crucial for integrating internal and external signals to orchestrate plant responses to both biotic and abiotic stresses.

Our previous research established that the transcription factor GmWRKY40 promotes soybean defense against *P. sojae* [31]. However, the precise molecular mechanism underlying its function remained to be elucidated. In this study, we explored this mechanism and discovered that *GmWRKY40* operates via a dual-action model within the nucleus. On one hand, it directly binds to and represses the promoter of *GmJAZ1*, a key repressor in the JA signaling pathway, leading to elevated endogenous JA levels and activation of JA-mediated defenses. On the other hand, we found that GmWRKY40 physically interacts with GmWRKY36, a negative regulator of *P. sojae* resistance. By elucidating this dual-regulatory model, our study addresses a significant research gap, providing novel mechanistic insights into how a WRKY transcription factor enhances the defense response by simultaneously engaging in antagonism with another WRKY protein and repressing a key repressor of the JA signaling pathway following infection by *P. sojae*. Furthermore, the discovery of this GmWRKY40-GmWRKY36 model highlights its breeding potential, providing excellent candidate genes for developing elite soybean cultivars with durable disease resistance.

## 2. Materials and Methods

### 2.1. Plant Cultivation and Pathogen Challenge

The soybean cultivars ‘Dongnong 50’ (susceptible, used for genetic transformation) and ‘Suinong 10’ (disease-resistant cultivar) were utilized in this study, alongside the predominant local strain *P. sojae* race 1 [32]. All materials were provided by the Key Laboratory of Soybean Biology of Chinese Education Ministry, which also isolated, identified and preserved the *P. sojae* strain. Soybean seeds were germinated in a substrate consisting of a 1:1 mixture of peat soil and vermiculite (Supplier: HONGLI, Harbin, China), which was sterilized by autoclaving at 121 °C for 2 h before use. Soybean seeds were germinated in soil and maintained in a growth chamber at 25 °C with 70% relative humidity under a 16 h light/8 h dark cycle, with a light intensity of 150 µmol m^−2^ s^−1^. *Nicotiana benthamiana* plants were grown at 25 °C with a 18 h light/6 h dark photoperiod, and *Arabidopsis thaliana* plants were grown at 23 °C with an 8 h light/16 h dark photoperiod.

*P. sojae* was cultured on V8 agar plates (10% (*v*/*v*) V8 juice, 0.2% (*w*/*v*) CaCO_3_, and 2% (*w*/*v*) agar) at 23 °C for 7 days. Zoospores were released from the plates and the concentration was adjusted to 1 × 10^5^ zoospores/mL using a hemocytometer. For the hairy root assays, a 10 µL aliquot of this zoospore suspension was applied directly to the root tip.

### 2.2. Soybean Hairy Root Transformation

The full-length GmWRKY36 gene was isolated by PCR using specific primers (Table S1), ligated into the pEAST-blunt vector (TransGen Biotech, Beijing, China), and its sequence was confirmed. All constructed plasmids were verified by Sanger sequencing (Sangon Biotech, Shanghai, China) to ensure sequence accuracy. All cloning PCRs were performed using 2× Phanta Flash Master Mix High-Fidelity DNA Polymerase (Vazyme, Nanjing, China). The general thermal cycling conditions were: 98 °C for 30 s, followed by 35 cycles of 98 °C for 10 s, 60 °C for 30 s, and 72 °C for 30 s/kb, with a final extension at 72 °C for 2 min. PCR products were verified by agarose gel electrophoresis. For functional characterization, this sequence was utilized to build overexpression and RNAi constructs within the pCAMBIA3301 and pFGC5941 backbones, respectively. The constructs were transformed into *Agrobacterium rhizogenes* strain K599. To generate transgenic hairy roots, the transformed *A. rhizogenes* was grown to an OD_600_ of 0.8–1.2. Soybean cotyledons were then wounded with a sterile scalpel and infected with the bacterial suspension for 20 min. After co-cultivation, the explants were placed in culture dishes and incubated at 25 °C. Hairy roots emerged from a callus ridge on the cotyledons approximately 3 weeks post-inoculation. This procedure was adapted from the established protocols of Graham et al., 2007 [33]. Verification of the transgenic hairy roots was then performed: overexpression lines were confirmed using quantitative PCR (qPCR), GFP fluorescence and Western blot using an anti-Myc antibody (1:5000 dilution; Abcam, cat no: ab56). The RNAi lines were verified by qPCR and with QuickStix strips for the bar protein.

### 2.3. QRT-PCR

Gene expression levels were measured by one-step qRT-PCR on a Roche LightCycler 96 System, utilizing a commercial kit from Vazyme (Vazyme, Nanjing, China) with SYBR Green for detection. Each reaction was performed in a 20 µL volume, containing 10 µL of 2 × SYBR Green Master Mix, 0.4 µM of each primer, and 100 ng of cDNA template. The optimal annealing temperature for each primer set was determined using gradient PCR, typically ranging from 55 to 60 °C. The amplification program consisted of an initial 5 min hold at 94 °C, followed by 45 cycles of 94 °C (30 s), annealing at the optimized temperature for 30 s, and 72 °C (40 s). After the final cycle, a dissociation curve was generated by ramping the temperature from 55 °C to 100 °C, followed by a 10 min cooling phase at 72 °C. The amplification efficiency for each primer set was confirmed by standard curve analysis (see representative image in Figure S1). Data analysis was performed using the 2^−ΔΔCt^ algorithm, where transcript levels were normalized against two reference genes: *GmEF1β* (NM_001248778) and *GmTUB4* (EV263740).

### 2.4. Subcellular Localization of GmWRKY40

Coding region of *GmWRKY40* was inserted into the pCAMBIA1302 vector, creating a C-terminal fusion with GFP. The resulting plasmid GmWRKY40-GFP along with a GFP control was independently co-transfected with the nuclear marker H2B-mCherry into Arabidopsis protoplasts. The transfection was performed using a PEG-mediated approach as described by Yoo et al. [34]. Specifically, protoplasts were transfected at a density of 2 × 10^5^ protoplasts/mL using a 40% (*w*/*v*) PEG4000 solution with an incubation time of 15 min. After 16 h of incubation, the distribution of fluorescence within the transformed protoplasts was visualized using a Leica TCS SP8 confocal microscope (Heidelberg, Germany). For GFP, an excitation wavelength of 488 nm and an emission window of 500–540 nm were used. For mCherry, an excitation wavelength of 561 nm and an emission window of 590–650 nm were used. Laser power was maintained at a low level (5–10%) to minimize phototoxicity.

### 2.5. RNA-seq and Metabolome Analysis

A dual omics approach was employed to characterize the effects of *GmWRKY40* overexpression, using hairy roots from both the transgenic (*GmWRKY40*-OE) and control (EV) lines. For the transcriptomic profiling, total RNA was extracted using TRIzol reagent (Invitrogen). For quality control, RNA purity was checked using a NanoPhotometer^®^ spectrophotometer (Implen, Munich, Germany) and RNA integrity was assessed with an Agilent 2100 Bioanalyzer system (Agilent Technologies, Waldbronn, Germany). Only samples with a 260/280 ratio between 1.9 and 2.1 and an RNA Integrity Number (RIN) > 8.0 were selected for library construction. High-throughput sequencing of cDNA libraries was then conducted by Metware Biotechnology Co. (Wuhan, China) via the Illumina platform. The resulting sequence reads were mapped to the reference soybean genome (available at https://phytozome.jgi.doe.gov/ accessed on 12 November 2025) with HISATv2.1.0. Differential expression analysis was performed using the DESeq2 (v1.22.1) package. Genes were identified as differentially expressed if they exhibited an absolute |log_2_(fold change)| > 1 and an adjusted *p*-value < 0.05 (*p*-values were adjusted for multiple testing using the Benjamini & Hochberg method). The functional significance of this gene set was interrogated through GO and KEGG pathway enrichment analyses, with *p*-values calculated using a hypergeometric test.

For the metabolomic analysis, samples were extracted with a 70% methanol solution. The analysis was performed on an AB4500 Q TRAP UPLC/MS/MS System coupled with an Agilent SB-C18 column (1.8 µm, 2.1 mm 100 mm). The mobile phases consisted of pure water with 0.1% formic acid (A) and acetonitrile with 0.1% formic acid (B), run with a detailed gradient program at a flow rate of 0.35 mL/min. Detection was performed using Multiple Reaction Monitoring (MRM) scans with key parameters including an ion source temperature of 550 °C and an ion spray voltage of +5500 V/−4500 V. As relative quantification was used for this widely targeted analysis, no internal standard was included. Features were considered significantly altered if they passed the dual criteria of a Variable Importance in Projection (VIP) score ≥ 1 from an OPLS-DA model and an absolute log2 (fold change) ≥ 1. These significant metabolites were then annotated and mapped using the KEGG Compound and Pathway databases, respectively. Finally, a Metabolite Set Enrichment Analysis (MSEA) was conducted to identify pathways that were statistically overrepresented, with significance determined by hypergeometric test *p*-values.

### 2.6. Quantification of Jasmonic Acid (JA)

The endogenous JA content in hairy roots from both *GmWRKY40*-overexpressing and empty vector (EV) control lines was measured. This quantification was achieved using High-Performance Liquid Chromatography–Mass Spectrometry (HPLC-MS), with the extraction and analysis procedures following the protocol detailed by Zhu et al. [35].

### 2.7. ChIP Assays

Chromatin immunoprecipitation (ChIP) was conducted using transgenic hairy roots expressing either an empty vector (EV) or p35S:*GmWRKY40*-Myc, following the procedure of Saleh et al. [36]. Briefly, tissue from 30-day-old hairy roots was cross-linked with 1% (*v*/*v*) formaldehyde under vacuum for 15 min. The reaction was quenched by adding glycine to a final concentration of 0.15 M, after which their nuclei were isolated. The chromatin was then sheared to an average fragment size of 200–500 bp using a Sonics VCX 130 sonicator (Sonics & Materials, Inc., New Haven, CT, USA) with 15 cycles of 30 s ON/30 s OFF at 30% amplitude. To minimize non-specific interactions, the resulting soluble chromatin was pre-cleared using an anti-Myc-tag Mouse mAb (Abcam, cat no: ab56, agarose conjugated). The specific immunoprecipitation was then carried out with a fresh aliquot of the same antibody, while a parallel immunoprecipitation using a non-specific IgG antibody was performed as a negative control to account for background binding. Following purification of the precipitated DNA, enrichment was quantified by qPCR using SYBR Premix ExTaq Mix (TaKaRa, Dalian, China). The final data are presented as relative binding, calculated as the ratio of immunoprecipitated DNA to input DNA (IP/input).

### 2.8. Dual-Luciferase Reporter Assay

To investigate whether GmWRKY40 functions as a transcriptional regulator of *GmJAZ1*, we utilized a transient expression assay in *N. benthamiana* leaves. Promoter of *GmJAZ1* was fused to the firefly luciferase (*LUC*) [37] to act as a reporter for transcriptional activity. This was co-infiltrated with an effector plasmid expressing *GmWRKY40*. The level of transcriptional activation was determined by normalizing the measured LUC activity against that of a co-expressed Renilla luciferase (Rluc) internal control.

### 2.9. Y2H Assay

To investigate a potential physical interaction between GmWRKY36 and GmWRKY40, we employed a yeast two-hybrid (Y2H) system. The complete coding sequence of GmWRKY36 was fused to the GAL4 activation domain in the pGADT7 (prey) vector, while the full-length GmWRKY40 was fused to the GAL4 binding domain in the pGBKT7 (bait) vector. The Y2HGold yeast strain was co-transformed with these bait and prey constructs. Initial selection of successful transformants was performed on synthetic drop-out medium lacking tryptophan and leucine (DDO). To test for protein interaction, these colonies were then spotted onto a high-stringency selective medium (QDO) containing X-α-gal. The plates were incubated for 3 days at 30 °C, and interaction was confirmed by yeast growth and α-galactosidase activity. Each combination was verified using two independent clones.

### 2.10. BiFC Assay

For the bimolecular fluorescence complementation (BiFC) assay, we constructed the recombinant vectors GmWRKY40-pSAT6^C^ and GmWRKY36-pSAT6^N^. These plasmids along with a vector expressing H2B-mCherry as a nuclear localization marker were co-introduced into Arabidopsis protoplasts via PEG-mediated transformation. The protoplasts were then examined with a Leica TCS SP2 confocal microscope to detect the reconstituted YFP fluorescence (indicating protein interaction) and the mCherry signal for nuclear co-localization.

### 2.11. SLCA

For the split luciferase complementation assay (SLCA), we constructed the recombinant vectors *GmWRKY36*-nLUC and *GmWRKY40*-cLUC. These constructs were individually transformed into Agrobacterium tumefaciens strain GV3101 and then co-infiltrated into *N. benthamiana* leaves for transient co-expression. At 72 h post-infiltration, the leaves were treated with a 1 mM D-luciferin solution containing 0.01% Triton X-100. After a 20 min dark incubation period to quench chlorophyll autofluorescence, the luminescence resulting from the reconstituted luciferase was captured and quantified using a Tanon 5200 chemiluminescence imaging system (Tanon, Shanghai, China).

## 3. Results

### 3.1. Analysis of Expression and Subcellular Localization of the Soybean Transcription Factor GmWRKY40

Based on sequence analysis, GmWRKY40 was identified as a member of the WRKY transcription factor family. Its 1619 bp full-length cDNA harbors a 993 bp open reading frame (ORF) that translates into a 330-residue protein containing a typical WRKY domain (Figure S2A). A comparative analysis involving phylogenetic and multiple sequence alignments demonstrated that the GmWRKY40 protein is highly conserved across the plant kingdom. It exhibits 70.92% to 99.68% sequence identity with numerous orthologues, namely GmWRKY40 (NP_001237392.2), GsWRKY40 (XP_028205063.1), AdWRKY40 (XP_015938143.1), AiWRKY40 (XP_016175403.1), Ap-WRKY40 (XP_027340388.1), CcWRKY40 (XP_020232263.1), GgWRKY53 (QFI57448.1), GmWRKY56 (NP_001237558.1), LaWRKY40 (XP_019457136.1), MpWRKY40 (RDX63723.1), QlWRKY40 (XP_030972723.1), SsWRKY40 (TKY48016.1), StWRKY40 (KAF7814504.1), TpWRKY40 (XP_045807019.1), TrWRKY56 (AWN02128.1), VaWRKY40 (XP_017423098.1), VrWRKY40 (XP_014502339.1), and VuWRKY40 (XP_027929832.1) (Figure S2B).

**Figure 1:**
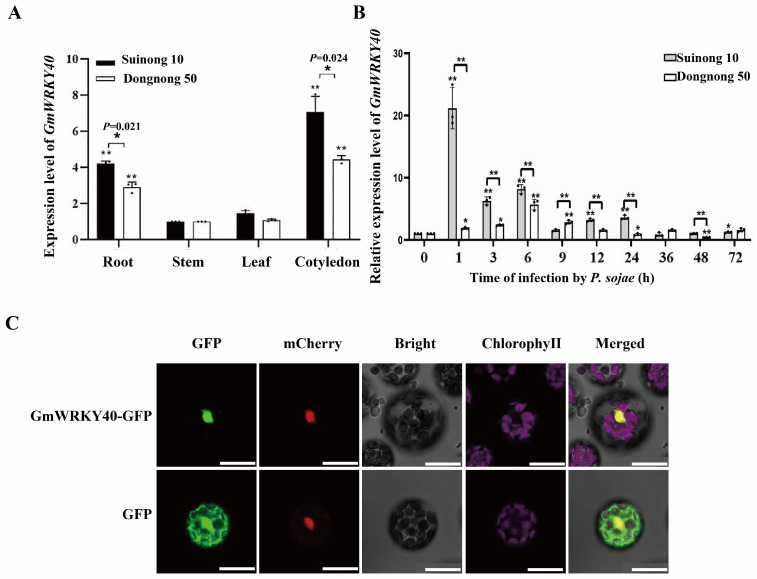
The expression profile and subcellular localization of GmWRKY40. (**A**) The Basal expression levels of GmWRKY40 in various tissues from the resistant (‘Suinong 10’) and susceptible (‘Dongnong 50’) soybean cultivars. (**B**) The time-course analysis of *GmWRKY40* transcript levels via qRT-PCR in both cultivars following *P. sojae* inoculation (0–72 hpi). *GmEF1β* and *GmTUB4* were used for normalization. Data are presented as mean ± SD (*n* = 3 biological replicates). The data were confirmed to follow a normal distribution. Statistical significance was determined by Student’s *t*-test (* *p* < 0.05, ** *p* < 0.01). (**C**) Subcellular localization of GmWRKY40. Confocal microscopy of Arabidopsis protoplasts co-expressing either GmWRKY40-GFP or a GFP control with the nuclear marker H2B-mCherry. Scale bar = 25 µm.

To characterize the expression dynamics of GmWRKY40, its transcript levels were quantified via qRT-PCR in two soybean cultivars with contrasting resistance to *P. sojae*: ‘Suinong 10’ (resistant) and ‘Dongnong 50’ (susceptible). The analysis showed a significantly higher basal expression of *GmWRKY40* in the tissues of ‘Suinong 10’ compared to ‘Dongnong 50’ (Figure 1A). Furthermore, *GmWRKY40* expression in ‘Suinong 10’ was strongly upregulated by *P. sojae* inoculation, peaking at 1 h and then rapidly decreasing—a response starkly contrasting with that of ‘Dongnong 50’ (Figure 1B). This indicates that the inducibility of GmWRKY40 by *P. sojae* is a distinctive feature of the resistant soybean cultivar.

To determine the subcellular localization of GmWRKY40, we co-expressed a *GmWRKY40*-GFP fusion construct with the nuclear marker H2B-mCherry. While cells expressing the GFP control vector exhibited diffuse fluorescence throughout the cell, the signal from the GmWRKY40-GFP fusion protein was confined exclusively to the nucleus. To confirm this localization is an intrinsic property of the protein and not a co-expression artifact, we expressed GmWRKY40-GFP alone and observed the same exclusive nuclear signal (Figure S3). This nuclear residency was confirmed by its clear co-localization with the H2B-mCherry marker (Figure 1C), demonstrating that GmWRKY40 is a nuclear-localized transcription factor.

### 3.2. GmWRKY40 Enhances the Activity of Antioxidant Enzymes in Response to P. sojae Infection

**Figure 2:**
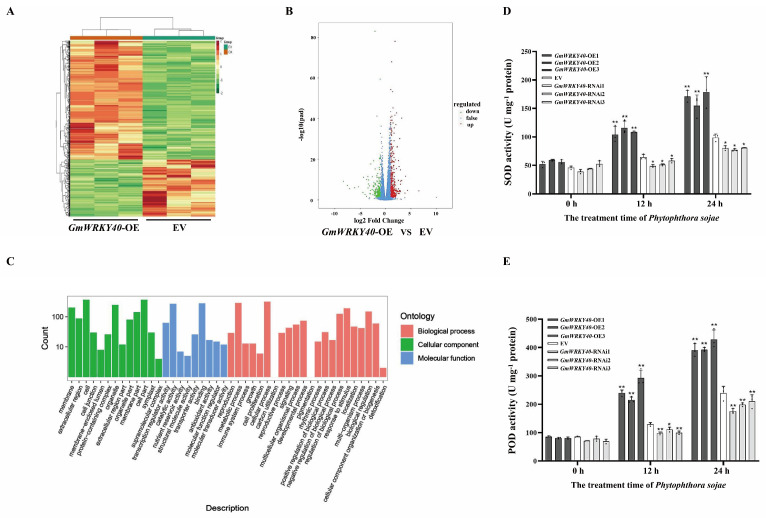
The transcriptomic and enzymatic analyses of transgenic soybean hairy roots with altered *GmWRKY40* expression. (**A**,**B**) The transcriptome analysis of *GmWRKY40*−OE versus empty vector (EV) control lines, showing (**A**) the heatmap of differentially expressed genes (DEGs) and (**B**) the volcano plot illustrating their distribution. (**C**) Gene Ontology (GO) functional enrichment analysis of the identified DEGs. (**D**,**E**) The time-course analysis of (**D**) SOD and (**E**) POD activities in *GmWRKY40*−OE, *GmWRKY40*−RNAi, and EV hairy roots following *P. sojae* inoculation. Data in (**A**−**E**) are from three biological replicates, with results for (**D**,**E**) presented as mean ± SD (*n* = 3 biological replicates). The data were confirmed to follow a normal distribution. Asterisks denote significant differences in (**D**,**E**) compared to the EV control (* *p* < 0.05, ** *p* < 0.01; Student’s *t*-test).

To further explore the disease resistance mechanism of GmWRKY40 and identify its potential downstream target genes, we generated GmWRKY40-OE and GmWRKY40-RNAi transgenic soybean hairy roots. These lines were successfully identified via a series of analyses, including observation of green fluorescence, Western blotting, PCR and qRT-PCR to confirm the intended alterations in gene expression (Figure S4). Subsequently, using three biological replicates for each line, we performed transcriptome sequencing (RNA-seq) analysis on the GmWRKY40-OE and EV transgenic hairy roots. Defining differentially expressed genes (DEGs) as those with an absolute |log_2_(fold change)| > 1 and an adjusted *p*-value < 0.05, this analysis identified 717 differentially expressed genes (DEGs) (fold-change > 2, FDR < 0.01), consisting of 484 up-regulated and 233 down-regulated genes (Figure 2A,B). GO functional analysis indicated that these DEGs are predominantly involved in stress response, growth and development, and hormone regulation processes, suggesting that GmWRKY40 may participate in these biological pathways in soybean (Figure 2C).

The analysis of the transcriptome data revealed that several differentially expressed genes (DEGs) were associated with peroxidase synthesis, prompting the hypothesis that *GmWRKY40* may be involved in regulating key antioxidant enzymes. To test this, we measured the activities of superoxide dismutase (SOD) and peroxidase (POD) in *GmWRKY40*-OE, *GmWRKY40*-RNAi and EV hairy roots at 0, 12, and 24 h post-inoculation (hpi) with *P*. *sojae*, which were chosen to represent the baseline, early, and mid-stage responses to infection. At 12 hpi, the activities of both SOD and POD were significantly higher in *GmWRKY40*-OE lines (* *p* < 0.01) and significantly lower in the *GmWRKY40*-RNAi lines (* *p* < 0.05) compared to the EV control. This trend was even more pronounced at 24 hpi (Figure 2D,E). Collectively, these findings indicate that the contribution of GmWRKY40 to *P. sojae* resistance is associated with the modulation of SOD and POD activities, two important components of the cellular antioxidant machinery.

### 3.3. Metabolomic Analysis Reveals the Involvement of GmWRKY40 in the Jasmonic Acid Pathway

**Figure 3:**
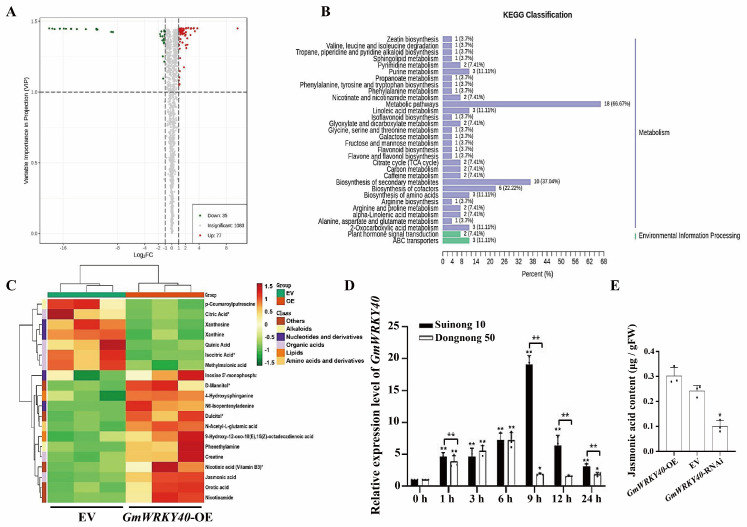
Metabolic and hormonal responses modulated by *GmWRKY40* in soybean. (**A**) The Volcano plot of differentially accumulated metabolites (DAMs) in *GmWRKY40*−OE vs. EV hairy roots. Red/green dots indicate metabolites with significant upregulation/downregulation (|log2FC| > 1, *p* < 0.05). (**B**) The KEGG functional annotation of DAMs, categorized by major metabolic pathways. (**C**) The hierarchical clustering heatmap of key stress-associated DAMs, with the color scale representing log2 (fold-change). (**D**) Transcriptional induction of GmWRKY40 by MeJA in leaves of resistant (‘Suinong 10’) and susceptible (‘Dongnong 50’) soybean cultivars (* *p* < 0.05, ** *p* < 0.01, Student’s *t*-test). (**E**) The endogenous JA content in transgenic hairy roots with altered *GmWRKY40* expression (OE, RNAi) compared to the empty vector (EV) control. Data in (**A**–**E**) are from three biological replicates, with results for (**D**,**E**) presented as mean ± SD (*n* = 3 biological replicates). The data were confirmed to follow a normal distribution. Asterisks denote significant differences in (**D**,**E**) compared to the EV control (* *p* < 0.05, Student’s *t*-test).

To further elucidate the metabolic mechanisms underlying GmWRKY40-mediated disease resistance, we performed a comparative metabolomic analysis on *GmWRKY40*-OE and EV transgenic hairy roots. Quality control analyses, including Total Ion Chromatogram (TIC) overlays and Principal Component Analysis (PCA), confirmed the high stability and reproducibility of the metabolomic data (Figure S5). From a total of 1195 identified metabolites, we screened for differentially accumulated metabolites (DAMs) using a threshold of |log2FC| > 1 and * *p* < 0.05. This analysis yielded 112 DAMs, comprising 77 up-regulated and 35 down-regulated compounds, many of which are associated with plant stress responses, such as flavonoids, organic acids, alkaloids and terpenoids (Figure 3A). KEGG enrichment analysis confirmed that these DAMs are involved in diverse metabolic pathways (Figure 3B). Furthermore, a clustering analysis of the most prominent pathways highlighted 20 key DAMs belonging to categories including alkaloids, nucleotides, lipids, amino acids and organic acids. Notably, JA was identified as a significantly altered metabolite, suggesting its potential role in the GmWRKY40-regulated defense response (Figure 3C). This conclusion was strongly supported by the significant accumulation of other key related metabolites, including its direct precursor 12-oxo-phytodienoic acid and its inactive storage form 5’-glucosyloxyjasmonic acid (Table 1). The coordinated enrichment of these functionally related compounds provides robust evidence that GmWRKY40 promotes a comprehensive activation of the JA biosynthetic and signaling pathway.

To investigate the interplay between GmWRKY40 and jasmonic acid (JA), a critical defense signaling molecule, we examined their regulatory relationship. First, we found that *GmWRKY40* expression is responsive to JA signaling; its transcription was induced by methyl jasmonate (MeJA) in both resistant (‘Suinong 10’) and susceptible (‘Dongnong 50’) cultivars, and direct comparison between them revealed a significantly stronger induction in the resistant line, particularly at 1, 9, 12, and 24 h (** *p* < 0.01) (Figure 3D). Reciprocally, we demonstrated that GmWRKY40 governs endogenous JA levels. Compared to EV, JA content was elevated in the *GmWRKY40*-OE hairy roots and significantly reduced in the *GmWRKY40*-RNAi lines (* *p* < 0.05) (Figure 3E). These findings establish that GmWRKY40 contributes to *P. sojae* resistance by promoting JA accumulation.

### 3.4. GmWRKY40 Regulates the Transcription of GmJAZ1

**Figure 4:**
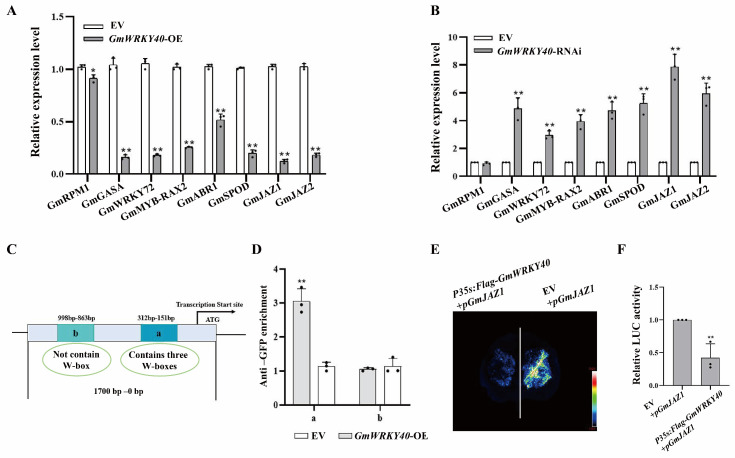
GmWRKY40 transcriptionally represses *GmJAZ1* via direct promoter binding. (**A**,**B**) The inverse expression patterns of stress-responsive genes in *GmWRKY40*-OE (**A**) and *GmWRKY40*-RNAi (**B**) hairy roots, determined by RT-qPCR. (**C**) Diagram of W-box elements within the *GmJAZ1* promoter, indicating the positions of ChIP-qPCR primers *GmJAZ1-a* (−151 to −312 bp) and *GmJAZ1-b* (−863 to −998 bp) relative to the transcriptional start site (TSS). (**D**) ChIP-qPCR reveals specific enrichment of GmWRKY40 at the *GmJAZ1* promoter. Data are presented as mean ± SD from three biological replicates. The data were confirmed to follow a normal distribution. Statistical significance was determined by Student’s *t*-test (* *p* < 0.05, ** *p* < 0.01). (**E**,**F**) The transient dual-luciferase assay in *N. benthamiana* shows that GmWRKY40 represses p*GmJAZ1*::LUC activity, evidenced by (**E**) luminescence imaging and (**F**) the quantitative LUC/REN ratio. Values are mean ± SD (*n* = 3). ** *p* < 0.01 (Student’s *t*-test).

Based on our RNA-seq data, we selected eight downregulated, stress-related genes to further investigate the regulatory role of *GmWRKY40*. RT-qPCR analysis confirmed that the expression of these genes (*GmJAZ1*, *GmJAZ2*, *GmWRKY72*, *GmSPOD1*, *GmRPM1*, *GmMYB-RAX2*, *GmGASA10* and *GmABR1*) was significantly suppressed in *GmWRKY40*-OE lines compared to the EV control, with *GmJAZ1* exhibiting the most substantial change (Figure 4A). Conversely, their expression was markedly upregulated following *GmWRKY40* knockdown in the RNAi lines (Figure 4B) (** *p* < 0.01 for both). These opposing results demonstrate that GmWRKY40 acts as a negative regulator of this specific set of stress-responsive genes, a finding that highlights the consistent repression of multiple key JA pathway components, including the core regulator GmJAZ1 and GmJAZ2, and the JA-responsive gene GmWRKY72.

WRKY transcription factors are known to regulate gene expression by binding to W-box motifs [(T)TGAC(C/T)] in the promoters of their targets [38]. A bioinformatic analysis revealed W-box elements in the promoters of all previously identified DEGs. Among these, *GmJAZ1* was selected for validation due to its strong repression and the presence of three W-boxes in its promoter (Figure 4C). To test for a direct interaction, we performed a Chromatin Immunoprecipitation (ChIP)-qPCR assay. The results showed a significant enrichment of GmWRKY40-MYC at the W-box-containing region (‘a’) of the *GmJAZ1* promoter, whereas no enrichment was observed at a control region (‘b’) lacking this motif (** *p* < 0.01) (Figure 4D). This demonstrates that GmWRKY40 directly and specifically binds to the *GmJAZ1* promoter in vivo.

To determine if GmWRKY40 directly regulates *GmJAZ1* transcription, we conducted a dual-luciferase reporter assay in *N. benthamiana* leaves. An effector construct expressing GmWRKY40-MYC was co-expressed with a reporter construct where the *GmJAZ1* promoter drove LUC expression (p*GmJAZ1*:LUC). Compared to the empty vector (EV) control, co-expression with GmWRKY40 significantly repressed the activity of the *GmJAZ1* promoter. This was evident from both the visibly reduced luminescence signal (Figure 4E) and the significantly lower LUC/REN activity ratio (Figure 4F), confirming that GmWRKY40 functions as a transcriptional repressor of *GmJAZ1*.

### 3.5. GmWRKY40 Interacts with GmWRKY36

**Figure 5:**
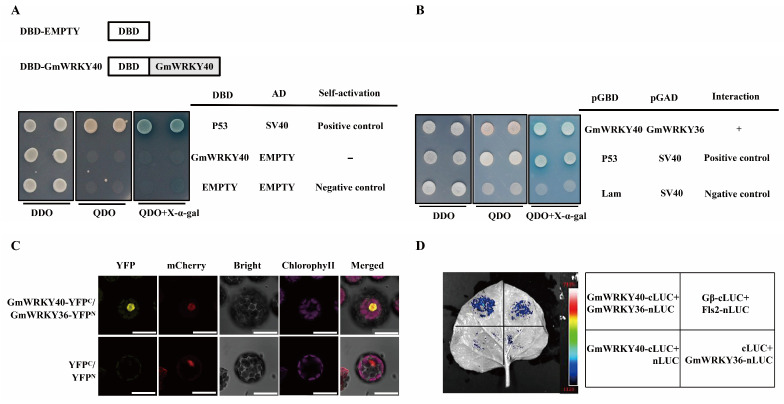
The Interaction of GmWRKY40 and GmWRKY36. (**A**) The analysis of self-activation activity of GmWRKY40. (**B**) The yeast two-hybrid analysis of GmWRKY40 and GmWRKY36. (**C**) The interaction between GmWRKY36 and GmWRKY40 was demonstrated by BiFC, using the YFPN/YFPC pair as a negative control (Scale bar, 25 µm). (**D**) The interaction between GmWRKY40 and GmWRKY36 was confirmed by SLCA, using the known Fls2-nLUC/Gβ-cLUC interaction as a positive control.

To identify proteins that interact with GmWRKY40, we first performed a yeast two-hybrid (Y2H) assay and confirmed that GmWRKY40 lacks transcriptional auto-activation, making it a suitable bait (Figure 5A). A subsequent Y2H screen of a soybean cDNA library of the *P. sojae*-resistant soybean cultivar ‘Suinong 10’ treated with *P. sojae* using GmWRKY40 as bait identified the transcription factor GmWRKY36 as an interacting partner (Figure S6). The direct interaction between GmWRKY40 and GmWRKY36 was then confirmed in a targeted Y2H assay, where co-expression of the two proteins enabled yeast growth on high-stringency selective medium (QDO/X-α-Gal), while negative controls failed to grow (Figure 5B).

This interaction was further validated in planta using two independent methods. First, a BiFC assay in Arabidopsis protoplasts revealed that co-expression of GmWRKY40-YFP^C^ and GmWRKY36-YFP^N^ generated a distinct YFP signal specifically localized to the nucleus (Figure 5C). Second, in a split luciferase complementation assay (SLCA) in *Nicotiana benthamiana*, co-expression of GmWRKY40-cLUC and GmWRKY36-nLUC successfully reconstituted luciferase activity, producing a strong luminescent signal (Figure 5D). Collectively, these results from Y2H, BiFC, and SLCA assays consistently demonstrate that GmWRKY40 physically interacts with GmWRKY36, and this interaction occurs within the nucleus of plant cells.

### 3.6. GmWRKY36 Promotes Soybean Susceptibility to P. sojae

**Figure 6:**
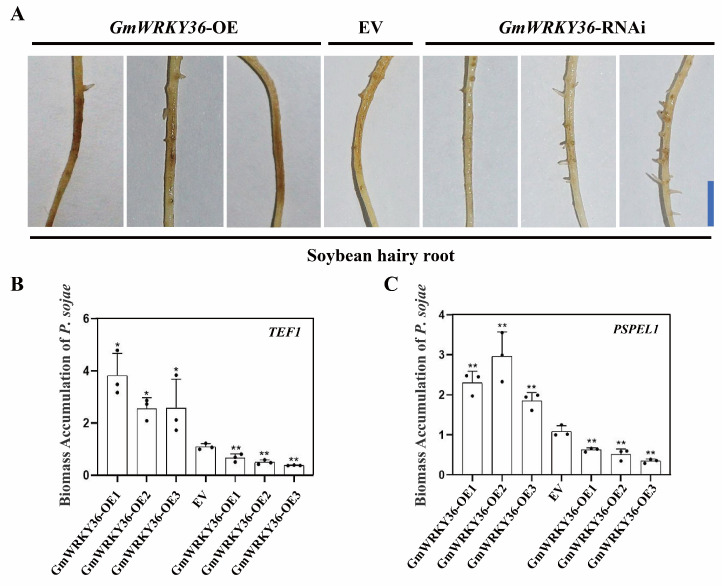
*GmWRKY36* negatively regulates soybean resistance to *P. sojae.* (**A**) The disease phenotypes of soybean hairy roots expressing an empty vector (EV), *GmWRKY36*-OE, or *GmWRKY36*-RNAi at 72 h post-inoculation (hpi) with *P. sojae*. The blue bar indicates the scale bar (0.5 cm). (**B**,**C**) The relative *P. sojae* biomass in the indicated roots, quantified by RT-qPCR using the fungal (**B**) *TEF1* and (**C**) *PSPEL1* genes as markers. Fungal gene expression was normalized to soybean *GmEF1β* and *GmTUB4*. Data are presented as mean ± SD (*n* = 3 biological replicates). The data were confirmed to follow a normal distribution. Asterisks denote significant differences from the EV control via Student’s *t*-test (* *p* < 0.05, ** *p* < 0.01).

To elucidate the function of *GmWRKY36* in response to *P. sojae*, we generated transgenic soybean hairy roots overexpressing *GmWRKY36* (OE) or silencing it via RNA interference (RNAi). Following successful validation of these lines (Figure S7), we inoculated them with *P. sojae*. At 72 hpi, the *GmWRKY36*-OE lines displayed exacerbated disease symptoms, such as severe soft rot, whereas the GmWRKY36-RNAi lines showed significantly attenuated symptoms compared to the empty vector (EV) control (Figure 6A). These visual phenotypes were corroborated by pathogen biomass quantification; *P. sojae* accumulation was significantly increased in *GmWRKY36*-OE roots but markedly decreased in *GmWRKY36*-RNAi roots relative to the EV (Figure 6B,C). Collectively, these results demonstrate that *GmWRKY36* negatively regulates soybean resistance to *P. sojae*.

## 4. Discussion

### 4.1. GmWRKY40 Promotes Soybean Defense Against P. sojae

Transcriptional reprogramming represents a fundamental defense mechanism in plants, wherein the WRKY families of transcription factors (TFs) are pivotal regulators [39]. Building on our previous work that identified GmWRKY40 as a WRKY TF repressed by GmLHP1 [31], this study sought to genetically and functionally characterize its role in disease resistance. Following *P. sojae* infection, *GmWRKY40* transcript abundance was significantly elevated in resistant soybean cultivar ‘Suinong 10’ compared to the susceptible cultivar ‘Dongnong 50’, exhibiting a positive correlation with the resistance phenotype (Figure 1B). Functionally, ectopic expression of *GmWRKY40* in transgenic soybean hairy roots not only conferred enhanced resistance to *P. sojae* but also potentiated the SOD and POD activities (Figure 2D,E). Collectively, these findings establish that *GmWRKY40* enhances soybean resistance to *P. sojae*, in part by augmenting the cellular ROS-scavenging machinery.

Phytohormone signaling networks are central to the orchestration of plant immunity [40,41,42]. While our prior research implicated the salicylic acid (SA) pathway in GmLHP1-mediated resistance [31], it is well-established that the SA pathway is predominantly effective against biotrophic pathogens. Conversely, defending against necrotrophic or semi-biotrophic pathogens such as *P. sojae* depends critically on activating the jasmonic acid (JA) signaling cascade [43]. Accordingly, our results showed that methyl jasmonate (MeJA) treatment significantly induced GmWRKY40 expression (Figure 3D). More definitively, overexpression of *GmWRKY40* led to a significant accumulation of endogenous JA in transgenic hairy roots (Figure 3E). These observations suggest that *GmWRKY40* positively regulates soybean resistance to *P. sojae* primarily through the modulation of the JA signaling pathway.

### 4.2. GmWRKY40 Activates the JA Signaling Pathway via Direct Transcriptional Repression of GmJAZ1

To dissect the molecular underpinnings of *GmWRKY40*-mediated JA signaling, we sought to identify its direct downstream targets. Transcriptomic profiling of hairy roots ectopically expressing *GmWRKY40* revealed significant transcriptional repression of *GmJAZ1*, which encodes a canonical negative regulator of the JA pathway, the JAZ (Jasmonate ZIM-domain) protein (Figure 4A,B). As repressors, JAZ proteins function as a crucial brake on the JA signaling cascade, and their regulation is paramount in mounting an effective immune response [44,45]. To validate a direct regulatory linkage between GmWRKY40 and *GmJAZ1*, we employed ChIP-qPCR and dual-LUC assays (Figure 4C–F). These assays confirmed that GmWRKY40 directly binds to the W-box *cis*-regulatory element within the *GmJAZ1* promoter, leading to potent transcriptional repression.

This discovery reveals how the transcription factor GmWRKY40, a positive regulator of resistance to *P. sojae*, activates defense signaling: it directly inhibits the expression of the negative regulator *GmJAZ1*, thereby de-repressing the JA signaling pathway. This mechanism provides a direct molecular basis for our observation of increased endogenous JA levels and offers new insights into the complexity of the WRKY-mediated regulatory network in soybean.

### 4.3. GmWRKY40 Forms an Antagonistic Module with the Negative Regulator GmWRKY36

Intricate regulatory networks formed by transcription factors are central to the fine-tuning of plant immune responses. Here, we demonstrate that GmWRKY40 physically interacts with another WRKY family member GmWRKY36.Y2H, BiFC and SLCA all confirmed the GmWRKY40 and GmWRKY36 interaction in the nucleus (Figure 5B–D). Functionally, *GmWRKY36* acts as a negative regulator of resistance to *P. sojae* (Figure 6), in direct contrast to the pro-resistance role of GmWRKY40.

The direct physical association between a positive (GmWRKY40) and a negative (GmWRKY36) regulator strongly implies a functional antagonism. We posit that this antagonism is manifested at the protein level, wherein GmWRKY36 heterodimerizes with GmWRKY40, thereby impeding or abrogating its DNA-binding affinity for target promoters, such as that of *GmJAZ1*. Such a mechanism of antagonistic regulation allows for the precise calibration of defense responses, preventing fitness costs associated with over-activation, a principle exemplified by the WRKY10-VQ8 interaction in rice heat tolerance [46].

**Figure 7:**
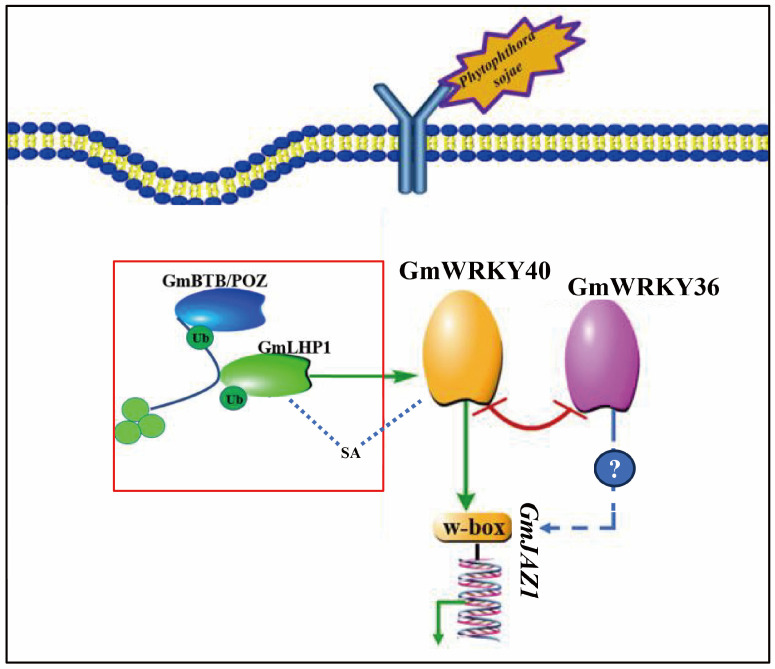
A proposed model for GmWRKY40-mediated resistance to *P. sojae* in soybean. This model illustrates the regulatory pathway mediated by GmWRKY40. The signaling module highlighted in the red box was established in our previous work [31]. In this initial model, the positive regulator GmBTB/POZ targets the transcriptional repressor GmLHP1 for degradation. This action de-represses *GmWRKY40*, enabling its crucial role in salicylic acid (SA)-dependent immunity against *P. sojae*. The present study expands this model by elucidating the downstream mechanisms of GmWRKY40 and identifying a new interacting partner. We demonstrate that upon induction by *P. sojae*, GmWRKY40 directly suppresses the expression of the key JA repressor *GmJAZ1* by binding to its promoter. This repression of *GmJAZ1* activates the JA signaling pathway, leading to higher endogenous JA levels and enhanced antioxidant enzyme activity, which contributes to a more robust defense response. Furthermore, we found that GmWRKY40 physically interacts with GmWRKY36, a susceptibility factor for *P. sojae* infection in soybean, suggesting a potential antagonistic relationship that fine-tunes the overall immune response. Taken together, our findings reveal a dual mechanism by which GmWRKY40 enhances resistance: activating the JA pathway via *GmJAZ1* suppression and engaging in a potentially antagonistic interaction with the negative regulator GmWRKY36 to modulate resistance to *P. sojae*.

Taken together, these findings lead us to propose the following model (Figure 7): Upon infection by *P. sojae*, GmWRKY40 promotes soybean resistance to *P. sojae* by enhancing antioxidant capacity and activating the JA signaling cascade through the targeted repression the expression of *GmJAZ1*. Concurrently, GmWRKY36 counteracts the pro-immunity function of GmWRKY40 via direct protein–protein interaction, thereby attenuating the overall defense output.

Regarding the precise molecular basis for this antagonism, our core hypothesis is that it is achieved through competitive inhibition. We propose that GmWRKY40 and GmWRKY36 form a heterodimer. This interaction is structurally critical, as we hypothesize the resulting complex has a greatly reduced affinity for the W-box cis-elements within the target gene promoters. Consequently, the formation of this heterodimer effectively competes with the ability of functional GmWRKY40 to bind to the W-box elements in the promoter region of target genes *GmJAZ1*. This provides a clear structural basis for how GmWRKY36 antagonizes the DNA-binding of GmWRKY40, thereby impairing its transcriptional repression capacity.

Future studies will be specifically aimed at validating this competitive inhibition hypothesis. Specifically, Electrophoretic Mobility Shift Assays (EMSAs) can determine if the GmWRKY40-GmWRKY36 heterodimer has a reduced binding affinity for W-box elements compared to GmWRKY40 alone. Furthermore, transient expression assays will be critical to assess whether GmWRKY36 can compromise the GmWRKY40-mediated repression of the *GmJAZ1* promoter in vivo, thus fully elucidating the functional outcome of this antagonistic regulatory module. Additionally, it is important to acknowledge the limitations of the hairy root system used in this study. This system primarily reflects local root immunity and has limitations in representing systemic immunity. Therefore, future studies using stable transgenic plants are necessary to validate these findings and to assess resistance to *P. sojae* at the whole-plant level.

## 5. Conclusions

This study reveals that *GmWRKY40* confers resistance to *P. sojae* in soybean by activating the jasmonic acid (JA) pathway through the direct repression of *GmJAZ1*, while also engaging in an antagonistic interaction with the *P. sojae*-negative regulator GmWRKY36. These findings elucidate a critical regulatory axis controlling disease resistance and provide key targets for engineering durable resistance in soybean.

## Figures and Tables

**Table 1:** Key Enriched Metabolites in the Jasmonic Acid (JA) Signaling Pathway.

Metabolite	Formula	Index	Class I
Jasmonic acid	C_12_H_18_O_3_	pme1654	Organic acids
5’-Glucosyloxyjasmanic acid	C_18_H_28_O_9_	Lmzn001582	Phenolic acids
12-Oxo-phytodienoic acid	C_18_H_28_O_3_	Zmyn004548	Lipids
13(s)-hydroperoxy-(9z,11e,15z)-octadecatrienoic acid	C_18_H_30_O_4_	Zmzn003953	Lipids
α-Linolenic Acid	C_18_H_30_O_2_	mws0367	Lipids
9-Hydroperoxy-10E,12,15Z-octadecatrienoic acid	C_18_H_30_O_4_	pmb2791	Lipids
Linoleic acid	C_18_H_32_O_2_	mws1491	Lipids

## Data Availability

All data are represented in the article and its Supplementary Materials.

## Supplementary Materials

The following supporting information can be downloaded at: https://www.mdpi.com/article/10.3390/biology14121769/s1, Table S1: List of primers used in this study; Figure S1: Representative melting curve for qRT-PCR primers. Figure S2: Nucleotide and amino acid sequences analyses of GmWRKY40; Figure S3: Subcellular localization of GmWRKY40. Figure S4: Identification of *GmWRKY40* transgenic soybean hairy roots; Figure S5: Quality control assessment of metabolomic data stability and reproducibility. Figure S6: Screening of interacting proteins of GmWRKY40 by yeast two-hybrid library; Figure S7: Identification of GmWRKY36 transgenic soybean hairy roots.
