# Supplementary material for: The WRKY Transcription Factor GmWRKY40 Enhances Soybean Resistance to Phytophthora sojae via the Jasmonic Acid Pathway
Source: Biology (Basel). 2025 Dec 11;14(12):1769. doi: 10.3390/biology14121769 (PMC12730507; doi:10.3390/biology14121769)
Supplement: Supplementary file 1 [file biology-14-01769-s001.zip › biology-4014396-supplementary/biology-4014396-supplementary.pdf]

Supplemental Table S1. List of primers used in this study.

Primers for quantitative real-time PCR and transcriptome sequencing analysis

Primer name	Primers (5'-3')
GmEF1ß -RT-qPCR	F: GGACTGCCGATGAACAAGATCATG
	R: ATGGAGATTAGACATGCGCTATAGC
GmTUB4-RT-qPCR	F: GGCGTCCACATTCATTGGA
	R: CCGGTGTACCAATGCAAGAA
GmWRKY40-RT-qPCR	F: TGCTTCTCTCACTTCACAAACTT
	R: TGTTTTCCACCTGCTTTTTGGG
GmJAZ1-RT-qPCR	F: TCAGTCTCTCCCACACACCCTT
	R: GTTTTGAGACATTCCCTTGCC
GmWRKY72-RT-qPCR	F: CTTCAACCCCAAGATTCCCTC
	R: TTTCTGCCAAAGCCTCCCT
GmSPOD1-RT-qPCR	F: ACTTTGGTTTAAGGCAAGAGGC
	R: TCGTCGGTTCGGAAGAATG
GmJAZ2-RT-qPCR	F: AACTTGCTTATCCTCCTTATCGC
	R: TGAACCTCTTGGCTCCTTGC
GmRPM1-RT-qPCR	F: TGTACGTATTTGGAGCAGGGG
	R: GAAAAGAAAAAGGCCACGAATGT
GmMYB-RAX2-RT-qPCR	F: AAGGGCATGGGTTGAAGTTAGA
	R: GGTGGAAGAAGGATTCAGGGT
GmGASA10-RT-qPCR	F: GGAACCTGCTACACTGATATGACC
	R: ATAGTAAACAGAATTGCACCCCAC
GmABR1-RT-qPCR	F: TTCTGCTTCGGGGTGATGTAA
	R: TGGATGGAAATGGTCGTGGT
TEF1	F: CCATGGTAATGGCGCCGAGGGATCAT
	R: ACTAGTAGCGATCTCATGCGGAGG
PSPEL1	F: CCGCGTACGTGGCTTTGGTGAG
	R: ATCTTGGCGACTGAGGCTGCTTAC

Primers for gene cloning

Primer name	Primers (5'-3')
GmWRKY36	F: ATGGCAAGAGGGGGTGGACTCT
	R: AACTATGACTAGTTCAATTT

Primers for constructs in transformation of soybean hairy roots and detection primer

Primer name	Primers (5'-3')
Bar	F: TTTCAATCCTAATAACAACGAGC
	R: ATGGACAACAACAGCCCTTC
GmWRKY36-RNAi1	F: TGCTCACCATCAGGATCC TTTGTTGTTGCTTGATGT
	R: CAAGCTTCGAATTCATGCTTCTCTCACTTCACAA
GmWRKY36-RNAi2	F: CTCACCATCAGGATCCTTTGTTGTTGCTTGATGT

	R: CGAGCTCAAGCTTCGAATTCATGGCAAGAGGGG
GmWRKY36-OE	F: GCACGCTGCCCAGGATCCATTATTATTGTGCAACA
	R: CGAGCTCAAGCTTCGAATTCATGGCAAGAGGGGGTGG

Primers for transient expression assay and yeast two-hybrid assays

Primer name	Primers (5'-3')
GmWRKY40-BD	F: GCCATGGAGGCCGAATTCATGCTTCTCTCACTTCACAA
	R: CGCTGCAGGTCGACGGATCCTTAATTATTATTGTGCAACA
pGADT7	F: TAATACGACTCACTATAGGGCG
	R: AGATGGTGCACGATGCACAG
GmWRKY36-AD	F: TACCAGATTACGCTCATATGATGGCAAGAGGGGGTGGACT
	R: TGCCCACCCGGGTGGAATTCTTATTTGTTGTTGCTTGATG

Primers for constructs in BiFC assays

Primer name	Primers (5'-3')
GmWRKY36-YFP^N	F: CGAGCTCAAGCTTCGAATTCATGGCAAGAGGGGGTGG
	R: TGCTCACCATCAGGATCC TTTGTTGTTGCTTGATGT
GmWRKY40-YFP^C	F: GCTCAAGCTTCGAATTC ATGCTTCTCTCACTTCACAA
	R: GCACGCTGCCCAGGATCCATTATTATTGTGCAACA

Primers for constructs in LCI assays

Primer name	Primers (5'-3')
GmWRKY36-nLUC	F: ACGAGCTCGGTACCCGGGATCCATGGCAAGAGGGGGTGG
	R: GGACGCGTACGAGATCTGGTCGACTTTGTTGTTGCTTGAT
GmWRKY40-cLUC	F: GGGGCGGTACCCGGGATCCCATGCTTCTCTCACTTCACAA
	R: CGAAAGCTCTGCAGGTCGACTTAATTATTATTGTGCAACA

Primers for subcellular localization

Primer name	Primers (5'-3')
GmWRKY40-1302	F: GGACTCTTGACCCATGGTTATGCTTCTCTCACTTCA
	R: CTCCTTTACTAGTCAGATCTATTATTATTGTGCAACA

Primers for ChIP assays

Primer name	Primers (5'-3')
GmJAZ1-a	F: GCCCCGAGGGATAGTCTTCT
	R: TGGTGCAAAGAGGTGACAGGT
GmJAZ1-b	F: AATAAAATAAAAATAATT
	R: GCATCGCCACCACATTATC

Primers for transient transcription dual-luciferase assay

Primer name	Primers (5'-3')
GmJAZ1-LUC	F: GGCCCCCCCTCGAGGTCGACGAGAAGCGATGAGTTT
	R: CTCTAGAACTAGTGGATCCAGGGTGTGTGGGAGAGAC

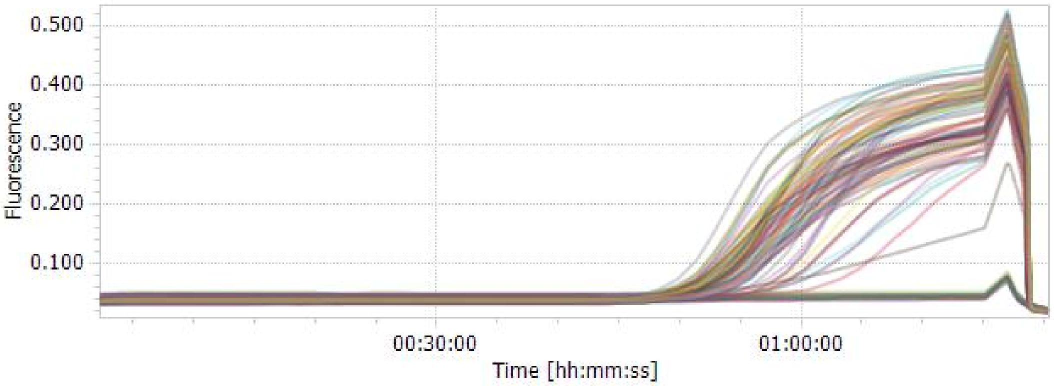

Figure S1. Representative standard curve for qRT-PCR primers. The curve was generated to validate the amplification efficiency and linearity for quantitative analysis.

A **B**

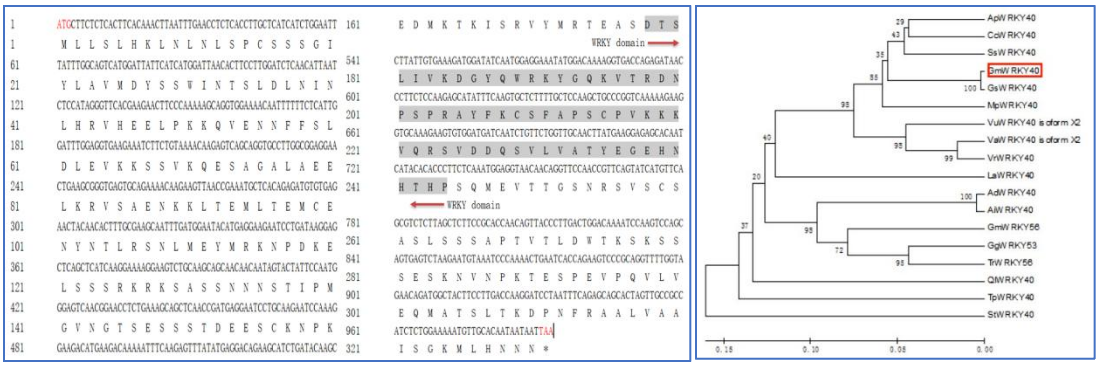

Figure S2. Nucleotide and amino acid sequences analyses of GmWRKY40. (A) The WRKY domain is shown in shadow. **(B)** Phylogenetic analysis of GmWRKY40 with orthologues from other plant species.

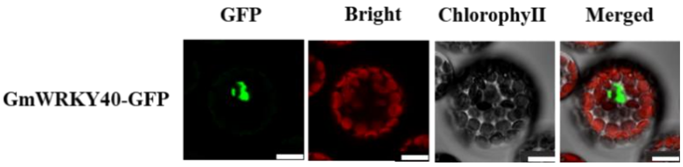

Figure S3. Subcellular localization of GmWRKY40. Confocal microscopy of Arabidopsis protoplasts expressing GmWRKY40-GFP. Scale bar = 10 µm.

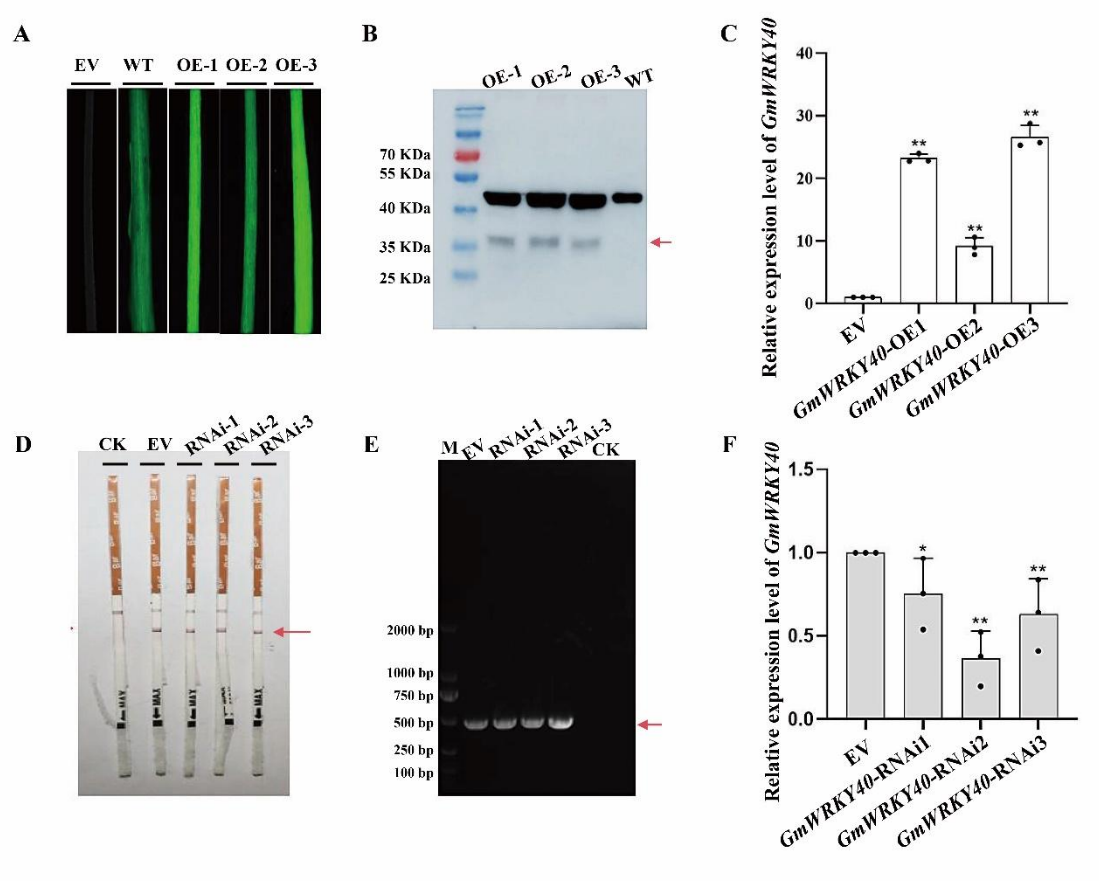

Figure S4. Identification of *GmWRKY40* transgenic soybean hairy root. **(A)** GFP fluorescence detection of the *GmWRKY40-OE* transgenic hairy roots and the empty vector (EV) controls.**(B)** Immunoblots showing the expression of the GmWRKY40-Myc fusion protein in three independent transgenic soybean hairy root and the wild-type (WT) controls. The total protein extracts were analyzed using a 12% SDS-PAGE and the immunoblot was probed with anti-Myc antibody. **(C)** Relative expression level of *GmWRKY40* in the *GmWRKY40-OE* plants and WT controls. **(D)** The three independent *GmWRKY40-RNAi* transgenic soybean hairy root were tested using QuickStix Kit for LibertyLink (bar) strips. **(E)** The *bar* in *GmWRKY40-RNAi* transgenic soybean hairy roots was detected by gel electrophoresis. **(F)** Relative expression level of *GmWRKY40* in the *GmWRKY40-RNAi* plants and WT controls.

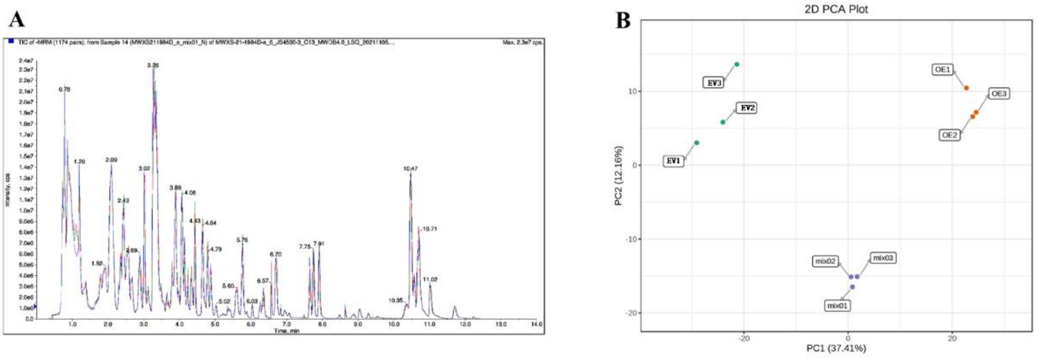

Figure S5. Quality control assessment of metabolomic data stability and reproducibility. **(A)** Overlaid Total Ion Chromatograms (TICs) from all samples. The consistent profiles demonstrate the stability of the analytical platform. **(B)** Principal Component Analysis (PCA) plot. The tight clustering of the quality control (QC) samples confirms the high reproducibility of the data.

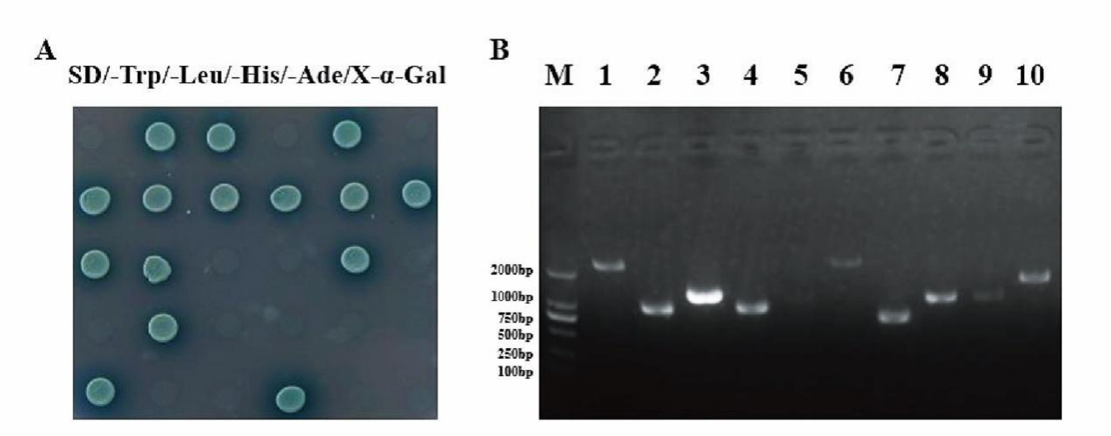

Figure S6. Screening of interacting proteins of GmWRKY40 by yeast two-hybrid library. **(A)** The interaction proteins were screened by SD/-Trp-Leu-His-Ade/X-α-Gal medium. **(B)** Cloning of GmWRKY40 candidate interacting protein fragments. M is the molecular weight standard of DL2000 DNA and 1-10 represent fragments of candidate interacting proteins.

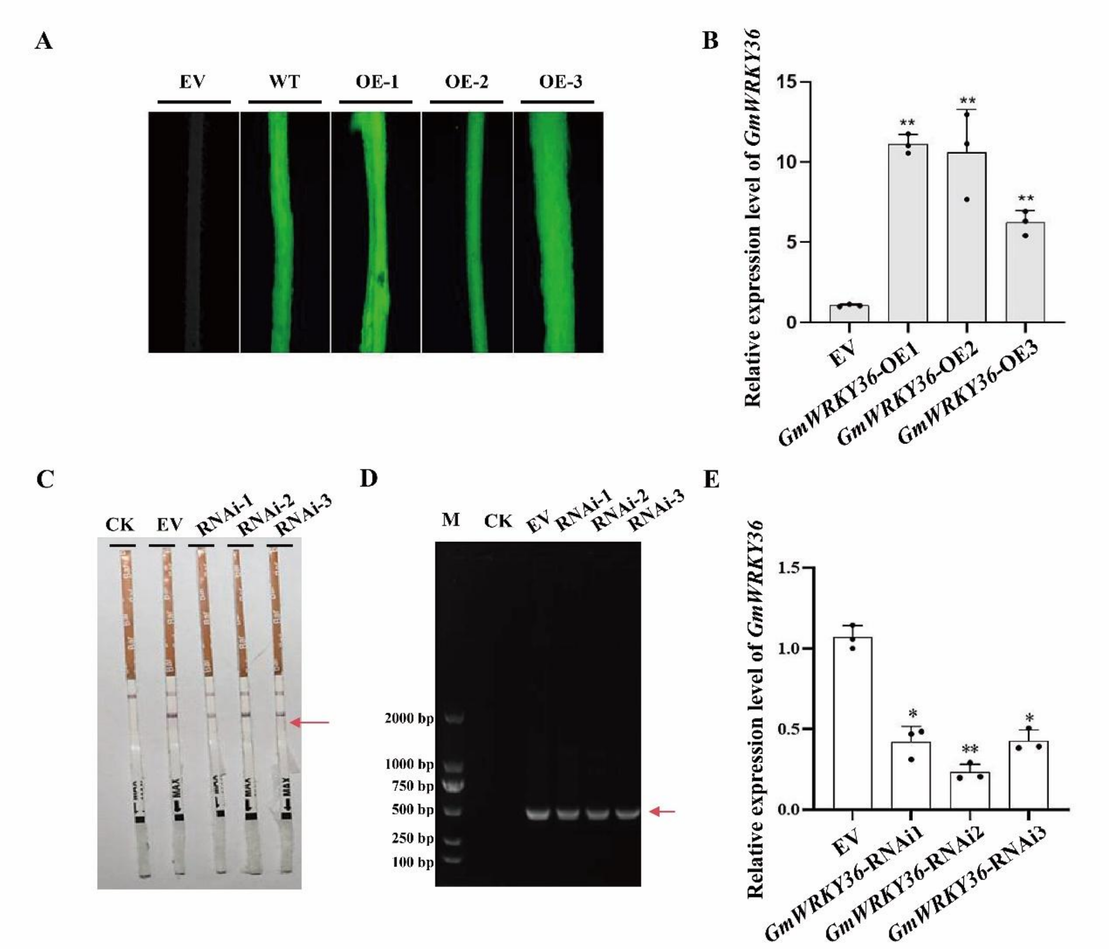

Figure S7. Identification of *GmWRKY36* transgenic soybean hairy root. **(A)** GFP fluorescence detection of the *GmWRKY36-OE* transgenic hairy roots and the empty vector (EV) controls. **(B)** Relative expression level of *GmWRKY36* in the *GmWRKY36-OE* plants and WT controls. **(C)** The three independent *GmWRKY36-RNAi* transgenic soybean hairy root were tested using QuickStix Kit for LibertyLink (bar) strips. **(D)** The *bar* in *GmWRKY36-RNAi* transgenic soybean hairy roots was detected by gel electrophoresis. **(E)** Relative expression level of *GmWRKY36* in the *GmWRKY36-RNAi* plants and WT controls.
